# Is being a night owl associated with higher migraine-related disability in patients with migraine?

**DOI:** 10.3389/fneur.2026.1812863

**Published:** 2026-05-25

**Authors:** Erkan Acar, Zeynep Özdemir, Pinar Yalinay Dikmen

**Affiliations:** 1 Department of Neurology, Acibadem Mehmet Ali Aydinlar University, School of Medicine, Istanbul, Türkiye; 2 Department of Neurology, Bakirköy Prof. Dr. Mazhar Osman Mental Health and Neurological Diseases Training and Research Hospital Neurology Clinics, Istanbul, Türkiye

**Keywords:** migraine, chronotype, circadian rhythm, sleep quality, migraine-related disability, eveningness, morningness

## Abstract

**Introduction:**

Emerging evidence suggests that circadian rhythms may influence migraine pathophysiology through interactions between sleep-wake regulation and pain modulation pathways. This study aimed to investigate the association between chronotype and migraine-related disability in patients with migraine.

**Methods:**

In this cross-sectional observational study, 200 patients with migraine and 110 healthy controls were included. Sleep quality was assessed using the Pittsburgh Sleep Quality Index (PSQI), chronotype was evaluated using the Morningness-Eveningness Questionnaire (MEQ), and migraine-related disability was measured using the Migraine Disability Assessment Scale (MIDAS). Episodic migraine (EM) and chronic migraine (CM) subgroups were also compared.

**Results:**

Migraine patients exhibited poorer sleep quality (PSQI: 7.28 vs. 4.37, *p* < 0.001), with chronic migraine (CM) patients showing the highest disability (MIDAS: 36.17 vs. 9.63, *p* < 0.001). No significant difference in chronotype distribution was observed between groups (*p* = 0.48). Morning chronotypes had lower MIDAS scores (16.95 ± 16.17) compared to intermediate (23.93 ± 22.28) and evening types (23.55 ± 20.85), though differences were non-significant (*p* = 0.082).

**Discussion:**

While chronotype does not directly correlate with migraine-related disability, poor sleep quality in migraine patients, particularly those with CM, underscores the need for sleep-focused interventions.

## Introduction

Circadian rhythms, which operate on a 24-h scale, are regulated by an internal biological clock and are believed to be generated by the suprachiasmatic nucleus (SCN) of the hypothalamus (1, 2). These rhythms include body temperature, various hormone levels, the number of immune cells in the blood, and the sleep–wake cycle.

Migraine pathophysiology is increasingly recognized as involving complex interactions between circadian regulation and pain modulation pathways. The SCN plays a central role in coordinating circadian rhythms and sleep–wake cycles while also contributing to trigeminovascular activation and migraine generation. Disruptions in circadian rhythms may influence migraine susceptibility by altering melatonin secretion, cortical excitability, and homeostatic regulatory mechanisms. Sleep deprivation can impair GABAergic inhibition and increase cortical glutamate levels, thereby facilitating cortical spreading depression, a key mechanism in migraine pathophysiology. In addition, overlapping neurotransmitter systems, including orexin, serotonin, dopamine, and norepinephrine, are involved in both sleep regulation and pain processing, further supporting a shared biological basis. These findings highlight that migraine is not only an episodic disorder but also closely linked to circadian and sleep–wake regulatory systems (3).

Chronotypes, or circadian typology—such as morning, intermediate, or evening types—have been recognized as a background variable in recent decades (4). According to an individual's chronotype, some people can be characterized as “early birds or larks” and some people as “night owls.” Early birds wake up early, go to bed early, and are more alert in the morning than in the evening. On the other hand, night owls prefer to wake up later in the day, are more alert at night, and go to bed late. These individuals tend to accumulate sleep debt during the week and extend their sleep duration on weekends.

Migraine is a highly prevalent and disabling disorder classified as a primary headache, and studies show that it is one of the leading causes of impairment worldwide (5). The relationship between chronotypes and migraine has not been clearly established. In a 2018 study, chronotypes and circadian timing in migraine were examined in 2,875 patients with migraine and 200 non-headache volunteers. The authors found that patients with migraine had an early chronotype compared to the control group (CG, 48.9% vs. 36.8%; *p* < 0.001). In this study, 40.2% of migraine patients reported that the onset of their attacks occurred in the early morning. However, Gori et al. (6) reported that both morning and evening types were more common in the migraine group (MG, *n* = 100) compared to control participants (*n* = 30). Moreover, 53% of migraine patients reported attack onset at night. Cevoli et al. (7) found that the distribution of chronotypes in 93 patients with menstrual migraine showed no differences compared to 85 healthy control participants. Finally, Viticchi et al. (8) assessed chronotype in migraine patients and the possible influences on the clinical expression of migraine (*n* = 100). They reported that chronotypes in migraine patients seemed to influence the number and duration of migraine attacks, but the intensity of migraine attacks was not influenced by chronotypes (8). Overall, only a limited number of studies with inconsistent results have been published to date.

In modern daily life, people often need to wake up early for work or school; therefore, the demands of modern life may be easier to meet for early birds. There are unresolved differences in chronotypes between migraine patients and healthy individuals. Whether someone is an early bird or a night owl for a patient with migraine may influence the level of migraine-related disability. This is particularly relevant because sleep disturbances are one of the primary triggers for migraine (9). Disturbed sleep patterns and insufficient sleep may lead to an increased frequency in migraine attacks for early birds with migraine (10). In fact, a higher prevalence of poor sleep quality has been reported in patients with migraine (11, 12), and improvement has also been observed after treatment (13). We hypothesized that a night owl chronotype in migraine patients may influence and increase migraine-related disability.

In this study, our first aim was to investigate differences in sleep quality and chronotypes between migraine patients and healthy individuals. Our second aim was to investigate whether being a night owl in migraine patients has a negative effect on migraine-related disability.

## Methods

This cross-sectional observational study included a total of 200 migraine patients who were recruited from the outpatient clinics of the neurology departments of two hospitals between January 2019 and December 2019. Neurologists established the diagnosis of migraine according to the International Classification of Headache Disorders, third edition (ICHD-III) (14).

The inclusion criteria for this study included patients between 18 and 60 years of age with a history of migraine for at least 6 months and who were willing to participate in the study.

Patients who presented with a headache of secondary nature, any unstable medical or psychiatric condition, who were pregnant, who were breastfeeding, or who were night shift workers were excluded. As this study was primarily based on self-assessed questionnaires, illiterate patients were not included.

The control group composed of relatives of patients who underwent a workup at our neurophysiology units for various peripheral disorders. Volunteers were evaluated by a neurologist, and individuals with a diagnosis of migraine or frequent tension-type headache were excluded. They were also excluded based on the exclusion criteria of the study.

A total of 264 patients with migraine and 154 volunteers were invited to participate in the study. Of these, 200 patients with migraine and 134 healthy individuals agreed to participate. Written informed consent was obtained from each participant following a detailed explanation of the study, and all participants provided their informed consent. The objectives and protocol of the study, which was conducted in accordance with the ethical principles stated in the “Declaration of Helsinki,” were approved by the Acibadem University Clinical Research Ethics Committee.

### Study measures sociodemographic data

To evaluate the sociodemographic characteristics of participants, both migraine patients and healthy individuals were asked to complete a questionnaire that collected information on age, sex, educational background, and occupation.

Migraine patients were questioned by a neurologist about the duration and clinical characteristics of their migraines, as well as family history, presence of comorbidities, precipitating factors, medication overuse, previous use of prophylactic treatment, and current treatments. The Migraine Disability Assessment Scale (MIDAS) was administered by neurologists.

All participants completed the Pittsburgh Sleep Quality Index (PSQI) and the morningness–eveningness questionnaire (MEQ) to evaluate sleep quality and chronotypes, respectively.

### PSQI

The PSQI is a questionnaire used to evaluate sleep quality over the previous month. It was developed by Buysse et al. (15). Its Turkish reliability and validity were established by Agargun et al. (16). The questionnaire consists of 19 self-rated questions and, if available, 5 additional questions rated by a bed partner or roommate. Only self-rated questions are scored and combined to produce seven subcomponent scores: subjective sleep quality, sleep latency, sleep duration, habitual sleep efficiency, sleep disturbances, use of sleep medication, and daytime dysfunction. These subcomponents yield a global score that ranges from 0 to 21. A score of >5 indicates poor sleep quality.

Higher scores are related to worse sleep quality.

### Chronotype assessment: MEQ

The MEQ was used to determine the chronotype of participants and assess whether their internal clock is more inclined toward being a night person (a “night owl”) or a morning person (an “early bird”). The questionnaire was first developed by Horne and Ostberg (17), and the Turkish reliability and validation were conducted by Punduk et al. (18). It contains 19 questions on sleep habits and fatigue. It categorizes patients into five groups, with lower scores associated with eveningness and higher scores with morningness. Scores range between 16 and 86. The total score is classified as follows: 16–30 (Definite Evening), 31–41 (Moderate Evening), 42–58 (Intermediate), 59–69 (Moderate Morning), and 70–86 (Definite Morning).

### MIDAS

The MIDAS is a questionnaire developed by Stewart et al. (19) and consists of five questions that quantitatively assess disability caused by migraine. The MIDAS score is calculated by summing the number of days of missed work or school, missed household chores, and missed non-work-related activities , as well as days at work or school and days of household chores in which productivity was reduced by 50% or more over the past 3 months due to migraine. The Turkish reliability and validation were conducted by Ertas et al. (20). The total score is graded as follows: Grade I (0 to 5 days), little or no disability; Grade II (6 to 10 days), mild disability; Grade III (11 to 20 days), moderate disability; and Grade IV (>21 days), severe disability.

### Statistical results

A total of 200 patients with migraine and 110 healthy volunteers were recruited for the study. The rates of episodic migraine (EM) and chronic migraine (CM) in our study were 53.5% (*n* = 107) and 46.5% (*n* = 93), respectively. Table 1 shows the demographic characteristics of all participants. Age and job status did not differ significantly between the two groups; however, the proportion of female individuals (81%) in the migraine group (MG) was higher than that in the control group (CG) (63.6%).

**Table 1:** Demographic characteristics of migraine patients and healthy controls.

Variable	All patients	Chronic	Episodic	Control	*P*-value
Number of patients	200	93	107	110	
Female (Sex) *N*	162 (81%)	76 (81.72%)	86 (80.37%)	70 (63.64%)	0.001^*^
Age (Mean)	34.49 ± 7.92	34.10 ± 7.56	34.84 ± 8.25	34.68 ± 10.63	0.478^†^
Job status					0.297^*^
Employee (*N*)	164	77	87	91	
Housewife	20	9	11	5	
Student	15	6	9	11	
Retire	1	1	0	3	

^†^Wilcoxon rank-sum test between the migraine and control groups. (The Wilcoxon test for age between episodic and chronic migraine patients showed a *p*-value of 0.373). ^*^Chi-squared test between the migraine and control groups. (The distribution of sex among all migraine patients and controls showed a *p*-value of < 0.05, indicating that sex and migraine status were not independent. The distribution of sex among episodic and chronic migraine patients had a *p*-value of 0.951. The distribution of job status among all migraine patients and controls showed a *p*-value of >0.05, indicating that job status and migraine status were independent).

Table 2 presents migraine features and MIDAS scores for all migraine patients, as well as EM and CM subgroups. In this study, 56.5% of migraine patients had a family history of migraine, and there was no statistical difference between EM and CM patients. Medication overuse headache (MOH) was reported by 33% of migraine patients. Indeed, MOH was significantly more common in CM patients (58%) compared to EM patients (11.2%) (*p* < 0.001).

**Table 2:** Clinical characteristics and MIDAS scores of migraine patients according to migraine subtype.

Clinical variables	All migraine Patients	Chronic	Episodic	*P-value*
Patient Number	200	93	107	
Migraine in Family History *(N)*	113	51	62	0.765^*^
MOH *N*	66	54	12	*p* < 0.001^*^
Migraine Duration (Mean)	10.70 ± 8.01	11.27 ± 8.14	10.21 ± 7.90	0.303^**^
MIDAS score (Mean)	21.97 ± 20.75	36.17 ± 21.09	9.63 ± 9.58	*p* < 0.001^**^
MIDAS A (Mean)	31.98 ± 27.37	52.89 ± 25.65	13.80 ± 10.86	*p* < 0.001^**^
MIDAS B (Mean)	7.32 ± 3.62	7.73 ± 5.03	6.96 ± 1.54	0.344^**^

^*^Chi-squared test between chronic and episodic migraine patients. (In this study, a family history of migraine was not dependent on whether patients had chronic or episodic migraine. The distribution of medication overuse headache (MOH) differed significantly between episodic and chronic migraine patients, with MOH being more common in patients with chronic migraine). ^**^Wilcoxon rank-sum test comparing scores between chronic and episodic migraine patients.

The mean duration of migraine among all patients was 10.70(±8.01) months, and there was no statistically significant difference between the two migraine subgroups (*p* = 0.303). The mean MIDAS score for all patients was 21.97 (±20.75), indicating a higher level of disability because of migraine. The MIDAS scores for CM and EM patients were 36.17 (±21.09) and 9.63(±9.58), respectively, and the difference between the two groups showed statistical significance (*p* < 0.001).

Headache days over the past 3 months also differed significantly between the EM and CM subgroups (13.80 vs. 52.89; *p* < 0.001).

However, pain intensity over the past 3 months, as reported by participants with EM and CM, did not show a statistically significant difference (7.7 vs. 6.96; *p* = 0.344).

Table 3 summarizes the comparison of total PSQI scores and its subcomponents between the MG and the CG, as well as between the two migraine subgroups. The sleep quality of the CG was not poor (4.37). However, the total PSQI score of patients showed poor sleep quality (7.28). Migraine patients exhibited significantly higher PSQI scores compared to controls (mean difference: 2.91, 95% CI: 2.24–3.57, *p* < 0.001), with a large effect size (Cohen's d = 0.83). The PSQI scores of the CM and EM groups were 8.24 and 6.45, respectively (*p* = 0.008). Comparison of the PSQI scores in both the CM group (8.24) and the EM group (6.45) showed a statistically significant difference compared to the CG (4.37) (*p* < 0.001). The PSQI subcomponents (subjective sleep quality, sleep latency, sleep duration, habitual sleep efficiency, sleep disturbances, sleep medicine use, and daytime dysfunction) showed statistically significant differences between the MG and the CG. Episodic migraine and CM patients also differed in the sleep disturbances subcomponent. Comparison of patients with CM and the CG showed a greater number of differences for all PSQI subcomponents between the two groups. The sleep latency, sleep duration, and use of sleep medication subcomponents of the PSQI did not show any statistically significant difference between patients with EM and the CG. The habitual sleep efficiency, sleep disturbances, and use of sleep medication subcomponents showed statistically significant differences between the CM and EM groups.

**Table 3:** Comparison of PSQI total and component scores between migraine patients and controls.

PSQI	All patients	Chronic	Episodic	Control
PSQI total score	7.28 ± 4.17	8.24 ± 4.45	6.45 ± 3.74	4.37 ± 1.73
Subjective sleep quality (C1)	1.35 ± 0.71	1.45 ± 0.71	1.26 ± 0.70	0.94 ± 0.47
Sleep latency (C2)	1.27 ± 0.96	1.37 ± 0.94	1.19 ± 0.97	0.86 ± 0.80
Sleep duration (C3)	0.92 ± 0.99	1.05 ± 1.01	0.81 ± 0.96	0.49 ± 0.71
Habitual sleep efficiency (C4)	0.72 ± 1.02	0.87 ± 1.09	0.60 ± 0.94	0.19 ± 0.48
Sleep disturbances (C5)	1.48 ± 0.74	1.69 ± 0.77	1.29 ± 0.66	1.08 ± 0.45
Use of sleep medication (C6)	0.31 ± 0.82	0.47 ± 1.03	0.17 ± 0.56	0.05 ± 0.27
Daytime dysfunction (C7)	1.23 ± 0.93	1.33 ± 0.97	1.13 ± 0.89	0.76 ± 0.69

Table 4 shows the chronotypes of the MG and CG based on MEQ scores, and there was no statistically significant difference between the two groups (*p* = 0.48). The intermediate sleep chronotype was the most common in both the migraine and control groups (61.5% vs. 54.5%). These results were also consistent when comparing patients with CM and EM (*p* = 0.140). Morning, intermediate, and evening chronotypes in the MG and CG were distributed as follows: Morning (*n* = 55, 27.5% vs. *n* = 35, 31.8%), intermediate (*n* = 123, 61.5% vs. *n* = 60, 54.5%), and evening (*n* = 22, 11% vs. *n* = 15, 13.7%)(*p* = 0.48). In the EM group, the distribution of chronotypes was morning (*n* = 35, 32.7%), intermediate (*n* = 63, 58.9%), and evening (*n* = 9, 8.4%). In the CM group, the distribution of chronotypes was morning (*n* = 20, 21.5%), intermediate (*n* = 60, 64.5%), and evening (*n* = 13, 14%). The distribution of the three chronotypes did not differ significantly between the EM and CM groups (*p* = 0.140).

**Table 4:** Distribution of chronotypes between migraine patients and healthy controls.

Groups	Evening	Intermediate	Morning
Migraine *N(%)*	22 (11%)	123 (61.5%)	55 (27.5%)
Control *N(%)*	15 (13.67%)	60 (54.55%)	35 (31.82%)
*P*-value^*^		0.484^*^	

^*^Chi-squared test for the distribution of sleep types between the migraine and control groups.

Table 5 demonstrates the total PSQI scores and PSQI subcomponents across the three chronotypes in the MG. *Post-hoc* analysis was performed only when significant *p*-values were observed among the three chronotypes in the MG. Total PSQI scores, as well as the subjective sleep quality, sleep latency, sleep disturbances, and daytime dysfunction subcomponents, showed significantly better results in the morning chronotype group.

**Table 5:** Comparison of PSQI scores among chronotypes in migraine patients.

Sleep parameter	Evening	Intermediate	Morning	P-value^*^
Subjective sleep quality (C1)	1.45 ± 0.59	1.43 ± 0.71	1.13 ± 0.72	0.026^*^
Sleep latency (C2)	1.32 ± 0.99	1.45 ± 0.95	0.85 ± 0.85	< 0.001^**^
Sleep duration (C3)	0.95 ± 1.04	0.97 ± 0.99	0.82 ± 0.98	0.558
Habitual sleep efficiency (C4)	0.54 ± 1.10	0.79 ± 1.03	0.65 ± 0.97	0.232
Sleep disturbances (C5)	1.50 ± 0.86	1.56 ± 0.70	1.28 ± 0.73	0.044^*^
Use of sleep medication (C6)	0.37 ± 0.79	0.38 ± 0.91	0.12 ± 0.58	0.066
Daytime dysfunction (C7)	1.55 ± 0.91	1.35 ± 0.94	0.82 ± 0.79	< 0.001^**^
PSQI	7.68 ± 3.98	7.93 ± 4.14	5.67 ± 3.95	0.002^**^

^*^indicates *p* < 0.05; ^**^indicates *p* < 0.01.

MIDAS scores in the MG based on chronotype did not show any statistically significant difference (Table 6). However, MIDAS scores differed significantly between the intermediate and morning chronotypes in the MG (*p* = 0.014), and MIDAS-A scores differed significantly between the evening and morning chronotypes in the MG (*p* = 0.025).

**Table 6:** Comparison of MIDAS scores among chronotypes in migraine patients.

MIDAS	Evening	Intermediate	Morning	*P*-value^*^
MIDAS	23.55 ± 20.85	23.93 ± 22.28	16.95 ± 16.17	0.082
MIDAS-A	42.73 ± 31.39	31.74 ± 25.95	28.22 ± 28.17	0.133
MIDAS-B	6.95 ± 1.13	7.07 ± 1.50	8.04 ± 6.48	0.681

^*^indicates *p* < 0.05.

A multivariable linear regression analysis was performed to evaluate the independent association between chronotype and MIDAS scores. Chronotype was not independently associated with MIDAS scores after adjusting for sex, migraine subtype, MOH, and sleep quality (Table 7). The strongest predictors of higher migraine-related disability were chronic migraine and MOH (*p* < 0.001 for both). The PSQI showed a trend toward significance (β = 0.45, *p* = 0.077).

**Table 7:** Multivariable linear regression analysis of factors associated with MIDAS scores.

Factors associated with MIDAS scores	Beta	*P*-value^*^
Evening vs. Morning	0.57	0.875
Intermediate vs. Morning	1.88	0.431
Female sex	0.20	0.939
Chronic migraine	17.75	< 0.001
MOH	16.71	< 0.001
PSQI	0.45	0.077

^*^indicates *p* < 0.05.

## Discussion

In our study, the migraine group (MG) showed poor sleep quality, with PSQI scores significantly higher in migraine patients. Although episodic migraine (EM) patients and the control group (CG) showed no difference in sleep quality, chronic migraine (CM) patients had the highest PSQI scores and experienced more daytime dysfunction than the CG. This finding suggests that migraine chronicity is associated with increasingly poor sleep quality.

Among chronotypes, the intermediate type was the most common in both the MG and CG. When comparing total PSQI scores and sub-scores among chronotypes in the MG, morning-type individuals demonstrated better sleep quality, shorter sleep latency, and better daytime function than intermediate and evening types. MIDAS scores were lowest among morning chronotypes. However, no statistically significant difference in migraine-related disability was found among the three chronotypes. The relationship between chronotype and migraine-related disability may be mediated by clinical factors, particularly sleep quality and disease severity.

As expected, CM patients had higher migraine-related disability and a higher frequency of MOH than EM patients. CM patients also experienced the most severe impact on sleep quality. Our findings align with those of Gori et al. (6), who found that sleep latency was the most affected sleep aspect in migraine patients. However, our results show that all PSQI sub-scores were worse in the MG compared to the CG, with CM patients experiencing the most severe sleep issues, including poorer habitual sleep efficiency, increased sleep disturbances, and greater use of sleep medication.

Comparison of sleep issues in EM and CM patients indicated that habitual sleep efficiency, sleep disturbances, and use of sleep medication were more common in CM patients. Sleep disturbances and sleep medication use, in particular, were the most frequently affected aspects of sleep among all migraine patients, likely contributing to daytime dysfunction in the CM group.

In contrast to the findings of Van Oosterhout et al. (21), who reported that early and late chronotypes were more prevalent among migraine patients than in non-headache controls, our results did not show a significant difference in chronotype distribution between the MG and CG, with the intermediate type being the most common in both groups. While Van Oosterhout et al. (21) used the Munich Chronotype Questionnaire, we used the MEQ, which might account for some of these differences.

Cevoli et al. (7), using the MEQ as well, reported findings similar to ours, with the intermediate type being the most common chronotype in migraine patients. Therefore, while there are some variations across studies, the distribution of chronotypes in migraine patients remains an area for further exploration.

Our study showed no statistically significant difference in migraine-related disability across the three chronotypes within the MG, although MIDAS scores indicated moderate disability in morning chronotypes and severe disability in intermediate and evening chronotypes.

While the difference in MIDAS scores between intermediate and morning chronotypes was statistically significant, differences among the other chronotype comparisons were not. Gori et al. reported that morning and evening types showed poorer sleep and higher migraine-related disability compared to the intermediate type (6), which is partly consistent with our findings. The findings of Viticchi et al. (8) similarly support the idea that chronotype may influence the frequency and duration of migraine attacks. Future studies should investigate the relationship between chronotype, sleep quality, and migraine attack frequency.

Our study has some limitations. First, the MG was predominantly composed of female individuals compared to the CG, which may have influenced our findings. Although patients with known psychiatric disorders were excluded clinically, subclinical symptoms may still have influenced both chronotype and migraine-related disability. Sleep disorders such as insomnia, obstructive sleep apnea, and restless legs syndrome, as well as psychiatric conditions that may act as confounding factors, were screened only through anamnesis. Sleep assessment was based solely on the PSQI, which does not fully capture the multidimensional nature of sleep disturbances such as sleep fragmentation, circadian misalignment, or objective sleep parameters. In addition, migraine attack frequency was not directly analyzed in the present study. This represents an important limitation, as a growing body of evidence indicates that sleep quality and long-term sleep hygiene play a significant role in modulating migraine attack frequency. Longitudinal data suggest that sustained poor sleep quality is associated with an increased migraine burden over subsequent weeks, highlighting its role as a risk factor rather than an immediate trigger. Therefore, including attack frequency analysis could have provided additional insights into the relationship between sleep-related parameters and migraine-related disability (22). The evening chronotype has been associated with higher rates of depression and poorer sleep quality, both of which may independently increase migraine-related disability. Therefore, residual confounding cannot be excluded. Another limitation is the imbalance in sex distribution between the migraine and control groups. The control group included relatives of patients evaluated in neurophysiology units, which may introduce selection bias. However, all controls were clinically evaluated to exclude primary headache disorders.

## Data Availability

The raw data supporting the conclusions of this article will be made available by the authors, without undue reservation.
